# Follow‐up of renal transplant recipients after acute COVID‐19—A prospective cohort single‐center study

**DOI:** 10.1002/iid3.509

**Published:** 2021-08-20

**Authors:** Nikolina Basic‐Jukic, Ivana Juric, Vesna Furic‐Cunko, Lea Katalinic, Josipa Radic, Zrinka Bosnjak, Bojan Jelakovic, Zeljko Kastelan

**Affiliations:** ^1^ Department of Nephrology, Arterial Hypertension, Dialysis and Transplantation, Clinical Hospital Centre Zagreb and School of Medicine University of Zagreb Zagreb Croatia; ^2^ Department of Nephrology, Clinical Hospital Centre Split and Faculty of Medicine University of Split Zagreb Croatia; ^3^ Department of Clinical and Molecular Microbiology Zagreb Croatia; ^4^ Department of Urology Clinical Hospital Centre Zagreb Zagreb Croatia

**Keywords:** complications, long‐COVID‐19, mortality, post‐COVID‐19, renal transplantation, SARS‐CoV‐2

## Abstract

**Introduction:**

Although most patients recover within several weeks after acute COVID‐19, some of them develop long‐lasting clinical symptoms. Renal transplant recipients have an increased mortality risk from COVID‐19. We aimed to describe complications occurring after COVID‐19 in this group of patients.

**Methods:**

A prospective single‐center cohort study was conducted at University Hospital Centre Zagreb. Patients with two negative reverse transcriptase‐polymerase chain reaction (RT‐PCR) tests for SARS‐CoV‐2 after COVID‐19 were eligible for further follow‐up at our outpatient clinic. They underwent detailed clinical and laboratory assessments. The primary outcome was the development of complications after COVID‐19.

**Results:**

Only 11.53% of renal transplant recipients who survived acute COVID‐19 were symptomless and free from new‐onset laboratory abnormalities during the median follow‐up of 64 days (range: 50–76 days). Three patients died from sepsis after discharge from the hospital. In 47 patients (45.2%), clinical complications were present, while 74 patients (71.2%) had one or more laboratory abnormalities. The most common clinical complications included shortness of breath (19.2%), tiredness (11.5%), peripheral neuropathy (7.7%), self‐reported cognitive impairments (5.7%), and dry cough (7.7%). Most common laboratory abnormalities included shortened activated partial thromboplastin time (50%), elevated D‐dimers (36.5%), elevated fibrinogen (30.16%), and hypogammaglobulinemia (24%). Positive RT‐PCR for cytomegalovirus (8.7%), Epstein–Barr virus (26%), or BK virus (16.3%). Multivariate analysis identified the history of diabetes mellitus and eGFR CKD‐EPI as predictors for the development of post‐COVID clinical complications. Six months after acute COVID‐19, elevated D‐dimers persisted with normalization of other laboratory parameters. Twenty‐nine patients were hospitalized, mostly with several concomitant problems. However, initially reported clinical problems gradually improved in the majority of patients.

**Conclusion:**

Post‐COVID‐19 clinical and laboratory complications are frequent in the renal transplant population, in some of them associated with significant morbidity. All patients recovered from acute COVID‐19 should undergo long‐term monitoring for evaluation and treatment of complications.

## Graphical abstract

Although most patients recover within several weeks after acute COVID‐19, some of them develop chronic injury of different organs or have nonspecific long‐lasting clinical symptoms. Renal transplant recipients have clinical and laboratory complications after acute COVID‐19.

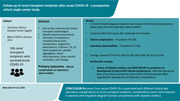

## 1 INTRODUCTION

While still focused on acute SARS‐CoV‐2 infection, which spreads in waves worldwide, many subjects have lingering illness following acute COVID‐19. This condition is known as “post‐COVID‐19” or “long COVID‐19”.^1^, ^2^, ^3^, ^4^ Although most patients recover within several weeks, some develop chronic injury of different organs, including lungs, heart, kidneys, brain, and other organs and tissues, or develop nonspecific long‐lasting clinical symptoms.^1^, ^2^, ^3^, ^4^, ^5^ Interestingly, some patients with severe COVID‐19 recover within a short period, while some with mild forms of the disease, or even asymptomatic, develop significant post‐COVID‐19 complications and require a long time for recovery.^6^ A few studies focused on post‐COVID‐19 in the general population.^1^, ^2^, ^3^, ^4^, ^5^, ^6^, ^7^, ^8^

Current literature suggests that hospitalized kidney transplant recipients have a high risk of death from COVID‐19.^9^, ^10^, ^11^, ^12^, ^13^, ^14^, ^15^, ^16^, ^17^, ^18^, ^19^ Due to their numerous comorbidities and immunocompromised state, it seems reasonable to expect more complications and prolonged recovery from COVID‐19. Much less is known about COVID‐19 in ambulatory treated renal transplant recipients, and, to our knowledge, there is no data on post‐COVID‐19 in this patients' group.

We aimed to describe complications occurring after COVID‐19 in renal transplant recipients from our transplant center.

## 2 METHODS

By the end of January 2021, we identified 140 adult kidney‐transplant patients with SARS‐CoV‐2 infection at University Hospital Centre Zagreb. Patients with two negative reverse transcriptase‐polymerase chain reaction (RT‐PCR) tests for SARS‐CoV‐2 were eligible for further follow‐up at our outpatient clinic. Patients followed in their local centers, and those with still positive RT‐PCR test for SARS‐CoV‐2 were excluded from the investigation.

A prospective observational cohort study evaluated the outcomes of 104 patients after the initial diagnosis of the COVID‐19.

A post‐COVID‐19 syndrome was defined as the presence of symptoms and laboratory abnormalities persisting beyond 8 weeks of the onset of acute COVID‐19 and not attributable to alternative diagnoses.

To assess clinical complications, patients were interviewed by a standardized survey by trained transplant nephrologists to recount symptoms during the acute illness and whether they persisted or some new occurred to assess clinical complications: fatigue, shortness of breath, cough, joint pain, headache, cognitive problems, intermittent fever, skin rash, hair loss or other specific problems.

They also underwent a detailed physical examination. Additional diagnostic methods were used individually (laboratory, radiologic). Data on immunosuppressive regimen and acute COVID‐19 characteristics were recorded. Venous blood samples were collected for complete blood count, biochemistry, coagulation examinations (prothrombin time (PT), activated partial thromboplastin time (APTT) and fibrinogen), D‐dimers, C3, C4, total complement, platelet aggregation with ADP (adenosine 5′‐diphosphate), serum electrophoresis, donor‐specific antibodies, and virology (molecular diagnostic detection for cytomegalovirus [CMV], Epstein–Barr virus [EBV], and BK virus [BKV]). Donor‐specific antibodies were determined by Luminex bead‐based technology (One lambda). Results were compared with historical values.

We had no data regarding the SARS‐CoV‐2 serology.

2.1

Any laboratory finding outside the reference ranges not present in the patient before the acute COVID‐19 was considered a laboratory complication. Any new onset clinical problem diagnosed by a history taking, physical examination, or radiologic assessment was considered a clinical complication. Allograft dysfunction was defined as the new onset increase in serum creatinine by 25% or newly developed proteinuria.

Patients have been in continuous follow‐up, with reassessment at six months after acute SARS‐CoV‐2 infection.

The primary outcomes included the presence of clinical complications or the occurrence of laboratory abnormalities.

Absolute and relative frequencies presented categorical data. The Shapiro–Wilk test tested the normality of the distribution of continuous variables. Continuous data were described by the median and the limits of the interquartile range (IQR). The Mann–Whitney *U* test was used to compare the median between two groups, while Fisher's exact test was used to analyze the differences between proportions. Logistic regression analysis was used to analyze the independent factors associated with clinical complications or laboratory abnormalities. A stepwise multivariable logistic regression was used to assess the association between potential risk factors and the development of laboratory or clinical complications, adjusting for known confounders. Variables assessed included demographic characteristics (i.e., age, gender, primary kidney disease), clinical characteristics (i.e., different comorbidities), acute COVID‐19 characteristics (i.e., presentation, need for hospitalization). Statistical significance in the univariate analysis was incorporated into the multivariate logistic regression model for in‐depth analysis. The level of significance was set at an *α* of .05. Considering the relatively small sample size and the possibility of overfitting in the multivariate logistic regression model, we adopted a stepwise forward method (probability for stepwise: entry, *p *< .05; removal, *p* < .1) for logistic regression analysis to reduce the number of independent variables entering the model. There was no substitution for the missing data. The statistical analysis was performed using MedCalc® Statistical Software version 19.6 (MedCalc Software Ltd.; https://www.medcalc.org; 2020) and the IBM SPSS Stat. 23 (IBM Corp., Released 2015, IBM SPSS Statistics for Windows, Version 23.0).

The study was approved by the University Hospital Centre Zagreb Ethics Committee.

## 3 RESULTS

### 3.1 Patients' characteristics

From March 2020 to January 2021, 140 patients who received renal allograft at Clinical Hospital Centre Zagreb developed COVID‐19 proven by positive SARS‐CoV‐2 real‐time RT‐PCR on the nasopharyngeal swab and were potentially eligible for investigation. Seventy‐seven patients required hospitalization (49 in intensive care unit), and 63 were treated in our outpatient clinic. Twelve patients (8.57%) required mechanical ventilation. Treatment included immunosuppression modification in 71 patients (68.2%), remdesivir (24 patients [17.1%]), hydroxychloroquine (12 patients [8.57%]), prophylactic use of low‐molecular‐weight heparin, increased doses of glucocorticoids and antibiotics. Additionally, eight patients (5.7%) received intravenous immunoglobulins, four (2.8%) received convalescent plasma, and ten patients (7.1%) received hyperimmune anti‐CMV globulin (in exchange for convalescent plasma). Two patients (1.4%) were treated with tocilizumab. Ten patients received no treatment while were diagnosed locally with the mild form of the disease and did not inform us timely about the infection.

**Figure 1:**
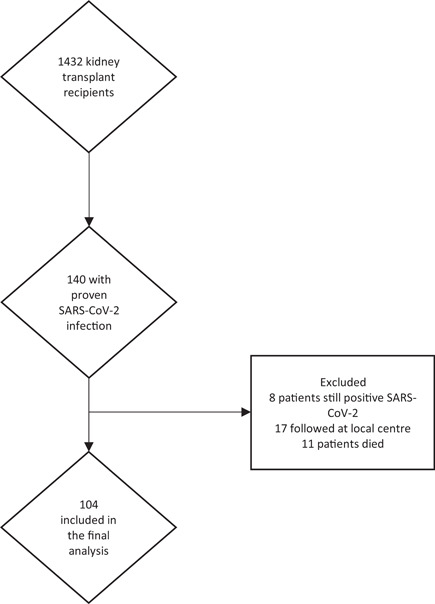
Flow‐chart of the study population

Out of the initial cohort of 140 patients with acute COVID‐19, 104 patients had detailed clinical and laboratory investigations. Eleven patients died (three from the acute myocardial infarction, other from the sepsis), eight patients had positive SARS‐CoV‐2 RT‐PCR test and 17 have still not been assessed at our clinic (Figure 1).

Patients have been assessed at our transplant outpatient clinic a median of 64 days (range: 50–76 days) after the initial diagnosis of the COVID‐19. Their characteristics are presented in Table 1.

**Table 1:** Patients' characteristics

Characteristic		Range
Gender [*n* (%)]		
Male	69 (66.3)	
Primary kidney disease [*n* (%)]		
Glomerulonephritis	32 (30.8)	
Diabetic nephropathy	7 (6.7)	
ADPKD	15 (14.4)	
Chronic pyelonephritis	9 (8.7)	
Nephroangiosclerosis	9 (8.7)	
Other	32 (30.8)	
Age (years) [median (IQR)]	56 (45–65)	24–80
Time from transplantation (months) [median (IQR)]	80 (42–126)	5–204
eGFR CKD‐EPI (ml/min/1.73 m^2^)	46 (34–61)	18–133
Proteinuria (g/day)	0.24 (0.13–0.5)	0.2–2.62
BMI (kg/m^2^)	27.1 (24.2–30.2)	18–45.8
Diabetes mellitus [*n* (%)]	21 (20.2)	
Hypertension [*n* (%)]	92 (88.5)	
Number of antihypertensive drugs [median (IQR)]	2 (1–3)	0–5
Previous thrombosis [*n* (%)]	7 (6.7)	
Previous myocardial infarction or stroke [*n* (%)]	11 (10.6)	
COVID‐19 initial symptoms [*n* (%)]		
Febrility	82 (78.8)	
Diarrhea	25 (24)	
Dyspnea	65 (62.5)	
Other	21 (20.2)	
Asymptomatic	10 (9.6)	
COVID‐19 initial complications		
Hospitalization	46 (44.2)	
Pneumonia	49 (47.1)	
Mechanical ventilation	1 (1)	
Other	25 (24)	
Initial immunosuppression		
Tac	70 (67.3)	
CyA	26 (25)	
Mikofenolat	94 (90.4)	
Aza	2 (1.9)	
Everolimus	13 (12.5)	
Decortin (doza) [medijan (IQR)]	5 (5–5)	0–20
Acute COVID‐19 treatment		
Cessation of MMF/Aza	35 (33.7)	
Decreasing MMF/Aza	36 (34.6)	
Cessation of Tac/CyA	1 (1)	
Decreasing Tac/CyA	19 (18.3)	
Hyperimmune anti‐CMV globuline	9 (8.7)	
Ivlg	4 (3.8)	
Remdesivir	17 (16.3)	
Hydroxychloroquine	3 (2.8)	

Abbreviations: Aza, azathioprine; ADPKD, autosomal dominant polycystic kidney disease; BMI, body mass index; CMV, cytomegalovirus; CyA, cyclosporine A; eGFR CKD‐EPI, estimated glomerular filtration rate Chronic Kidney Disease Epidemiology Collaboration; Ivig, intravenous immunoglobuline; tac, tacrolimus.

### 3.2 Post‐COVID‐19 clinical complications

In the short follow‐up, one or more rehospitalizations were necessary for 17 patients (16.3%) with one or more clinical problems (10 had recurrence of pneumonia (one with lung embolism, one with concomitant allograft dysfunction), five acute allograft dysfunction ± proteinuria, and two developed sepsis). Three patients died from sepsis during the post‐COVID‐19 follow‐up.

Clinical complications were present in 47 patients (45.2%) and included shortness of breath (20 patients [19.2%]), tiredness (12 patients [11.5%]), peripheral neuropathy (8 patients [7.7%]), self‐reported cognitive impairments (6 patients [5.7%]), dry cough (8 patients [7.7%]), worsening of hypertension (4 patients [3.8%]), deep venous thrombosis (3 patients [2.9%]), de novo diabetes mellitus (4 patients [3.8%]), skin changes (3 patients [2.9%]), hair loss (2 patients [1.9%]), dizziness (4 patients [3.8%]), costochondritis (2 patients [1.9%]) and anasarca (2 patients [1.9%]), while herpes zoster, lung embolism, paroxysmal atrial fibrillation, significant body weight loss (more than 10% of body mass), cardiorenal syndrome and spontaneous retroperitoneal bleeding were found in one patient each. Biopsy findings revealed borderline acute allograft rejection in one patient, while two had chronic active antibody‐mediated rejection. Most patients with long‐term post‐COVID‐19 symptoms had more than one clinical complication.

The bivariate analysis identified five significant predictors for the development of post‐COVID clinical complications. The strongest was hospitalization for acute disease, diabetes mellitus, and concomitant laboratory complications, while better allograft function estimated by CKD‐EPI decreased the probability of clinical complications. Stepwise multivariate regression analysis was used to examine the predictors significant for the prediction of clinical complications. Two predictors (diabetes mellitus and eGFR CKD‐EPI) had a unique statistically significant contribution to the model. The model was entirely statistically significant (*χ* ^2^ = 12.6, *p* = .002), and explained from 13.9% (according to Cox and Snell^20^) to 18.5% (according to Negelkerke^21^) variance in the presence of clinical complications, and correctly classified 60% of cases (Table 2).

**Table 2:** Bivariate and multivariate analysis used to examine predictors of clinical complications

	*β*	Wald	*p*	OR (95% CI)
Bivariate				
Diabetes mellitus	1.24	3.98	.04	3.44 (1.02–11.6)
Laboratory complication	1.48	4.38	.04	4.4 (1.09–17.6)
Allograft function (eGFR CKD‐EPI)	−0.03	6.1	.01	0.97 (0.94–0.99)
Hospitalization	1.24	7.02	.008	3.44 (1.38–8.58)
Fibrinogen	0.52	6.12	.01	1.69 (1.11–2.56)
Multivariate				
Diabetes mellitus	1.48	4.76	.03	4.42 (1.16–16.8)
Allograft function (eGFR CKD‐EPI)	−0.03	7.03	.008	0.97 (0.94–0.99)
Constant	1.54	5.66	.02	

Abbreviations: CI, confidence interval; eGFR CKD‐EPI, estimated glomerular filtration rate Chronic Kidney Disease Epidemiology Collaboration; OR, odds ratio.

The number of rehospitalizations has increased at 6 months post‐COVID‐19. Twenty‐nine patients were hospitalized, mostly with several concomitant problems (leucopenia, viral reactivation, sepsis, pneumonia, urinary tract infections, diabetes mellitus, rhabdomyolysis, psychotic reaction). Initially reported clinical problems gradually improved in the majority of patients. At 6 months, 4 patients (3.8%) still feel tired, 2 (1.9%) had peripheral neuropathy, 5 (4.8%) had diabetes requiring insulin treatment, and 4 (3.8%) complained of hair loss. Seven patients have still been followed by pulmologists with significant improvement of lung status both clinically and radiologically.

### 3.3 Post‐COVID‐19 laboratory abnormalities

At the initial evaluation, one or more laboratory abnormalities were present in 74 patients (71.2%). Although allograft function remained stable after acute COVID‐19 in most patients, 8 had allograft dysfunction (7.7%), in 6 of them with proteinuria. One required temporary dialysis post‐COVID‐19 but returned to initial allograft function, and two patients remained dialysis‐dependent (1.9%).

Laboratory abnormalities included increase in serum creatinine (8 patients [7.7%]), de novo development or worsening of proteinuria (6 patients [5.7%]), increased liver chemistries (2 patients (1.9%)), development of de novo DSA (6 patients [5.7%]), shortened APTT (52 patients [50%]), increased D‐dimers (38 patients [36.5%]; in 21 [20.2%] of them were 2× above the reference range), increased fibrinogen (29 patients [30.16%]), positive real‐time polymerase chain reaction for CMV (9 patients [8.7%]), EBV (27 patients [26%] patients) or BK virus (17 patients [16.3%], positive urine and/or blood test). Hypogammaglobulinemia was present in 25 patients (24%). C3 was decreased in 9 patients (8.65%), C4 elevated in one patient (0.9%), while total complement activity remained within the normal range in all patients. Platelet aggregation and prothrombin time were within normal range except for three patients treated with warfarin and had shortened PT. Anemia worsened in 17 patients (16.3%), eight patients (7.7%) had leukopenia, and nine thrombocytopenia (8.7%).

Six months after acute COVID‐19, d‐dimers remained elevated in patients who presented after the initial infection with values 2× above the reference range (21 patient), indicating either ongoing inflammation, coagulopathy, or both. Acetilsalycil acid (75 mg) was introduced in their treatment at the initial visit. Patients with elevated d‐dimers had longer transplant vintage compared with patients with normal values (median 104 months [range: 62–154] vs. 72 months [range: 31–118], *p* = .04). Activated partial thromboplastin time remained shortened in only two patients.

Two months after acute COVID‐19, we have introduced erythropoiesis‐stimulating agents in patients with anemia, which resulted in improved serum hemoglobin levels.

Over the observed period of 6 months, seven patients underwent indication biopsy, and one had allograft nephrectomy. One case of collapsing focal segmental glomerulosclerosis was recorded (the paper has been submitted for review). Two patients are dialysis‐dependent, and two have reached the preterminal stage of chronic allograft nephropathy without dialysis.

Viral reactivations remained a significant problem at six months post‐COVID‐19. Epstein Barr reactivation was recorded in 27% of patients and required decreasing the dosage of mycophenolate mofetil in 10 patients with a high number of copies (above 50,000/ml) and intravenous immunoglobulins (0.5 g/L) in 4 patients with a high number of copies accompanied with hypogammaglobulinemia. This group of patients is under close surveillance. CMV reactivation was severe in one patient with development of CMV colitis and resistance to ganciclovir and valganciclovir. She received foscarnet and continued with letermovir for secondary prophylaxis with hyperimmune anti‐CMV globulins. BK virus reactivations were more frequent in patients with lower C3 (median: 1.11 [interquartie range [IQR]: 0.93–1.33] vs. 1.37 (IQR: 1.1–1.44, *p* = .02) and C4 (median 0.21 [IQR: 0.16–0.25] vs. 0.27 [IQR: 0.2–0.3], *p* = .004).

We further investigated predictors for the development of laboratory abnormalities. Bivariate regression analysis recognized four significant predictors, the strongest being the existence of clinical complications. Decreasing the dose of tacrolimus during the acute SARS‐CoV‐2 infection had a protective effect (Table 3), remaining significant in the multivariate analysis.

**Table 3:** Bivariate and multivariate analysis used to examine predictors of laboratory abnormalities after COVID‐19

	*β*	Wald	*p*	OR (95% CI)
Bivariate				
Age	0.05	4.55	.03	1.05 (1.004–1.11)
eGFR CKD‐EPI	−0.03	4.24	.04	0.97 (0.95–0,99)
Decreasing Tac dose	−1.95	7.92	.005	0.14 (0.04–0.55)
Clinical complication	1.48	4.39	.04	4.4 (1.09–17,6)
Multivariate				
Decreasing Tac dose	−1.92	7.64	.006	0.15 (0.04– 0,57)
Constant	2.43	27.2	<.001	

Abbreviations: CI, confidence interval; eGFR CKD‐EPI, estimated glomerular filtration rate Chronic Kidney Disease Epidemiology Collaboration; OR, odds ratio; Tac, tacrolimus.

## 4 DISCUSSION

Published studies have focused on symptoms and outcomes of acute disease in renal transplant recipients with COVID‐19.^9^, ^10^, ^11^, ^12^, ^13^, ^14^, ^15^, ^16^, ^17^, ^18^, ^19^, ^22^, ^23^ To our knowledge, this is the first study focused on the post‐COVID‐19 outcomes in this group of patients.

We found that only 11.53% of renal transplant recipients who survived acute COVID‐19 had no clinical symptoms or were free from any laboratory abnormality during the median follow‐up of 64 days (range: 50–76 days). Prolonged symptom duration and clinical complications were present in 47 patients (45.2%), while 74 patients (71.2%) had one or more laboratory abnormalities. An Italian study found that in the general population of patients who recovered from COVID‐19, only 12.6% were completely free of any COVID‐19‐related symptom 60 days after the onset of infection.^5^ Delayed return to usual health^2^ and decreased health‐related quality of life have been reported.^3^ In the study of Huang et al., which included 1733 discharged patients with COVID‐19, the most common symptoms were fatigue or muscle weakness (63%) and sleep difficulties (26%).^4^ Moradian et al. reported that 42% of their patients were symptom‐free, while fatigue persisted in 19.5%, followed by dyspnea (18.5%), weakness (18%), and activity intolerance (14.5%).^8^

The proportion of patients reporting persistent symptoms in our study was similar or even lower than in other studies.^24^, ^25^, ^26^, ^27^, ^28^, ^29^, ^30^, ^31^, ^32^ I may be due to the long‐term history of significant clinical problems present in the renal transplant population used to different complications (especially anemia‐related) and may underreport fatigue and dyspnea, the most common prolonged post‐COVID‐19 symptoms in the general population. However, our patients developed numerous severe complications, rare in the general population and maybe kidney transplant‐specific. Additionally, our analysis included both hospitalized and patients treated in the outpatient clinic, while most studies mainly focused on hospitalized patients. A few studies of patients who were not hospitalized for COVID‐19 exist for comparison. The major problem is a very diverse symptom evaluation after the acute disease that disables precise comparison between different studies. In line with this problem, Stavem et al. reported that 53% of nonhospitalized women and 67% of men were symptom‐free 1.5–6 months after the acute COVID‐19. The need for hospitalization during the acute COVID‐19 was a significant adverse risk factor for developing clinical complications in our cohort. Their cohort included a wide time frame after COVID‐19^33^ with the expected decrease in occurrence and severity of post‐COVID‐19 symptoms.

In this study, hospitalization, diabetes mellitus, and renal allograft function estimated by eGFR CKD‐EPI, increased fibrinogen, and presence of any laboratory abnormality were predictors of clinical complications. In contrast, age, eGFR CKD‐EPI, and occurrence of clinical complications predicted the development of laboratory abnormalities. Published data indicates that people over age 50 and those with two or three chronic illnesses are more likely to develop the post‐COVID syndrome. Also, patients with very severe forms of acute disease were found to have an increased risk for post‐COVID.^1^ Preexistent chronic renal disease was identified as a risk factor for acute kidney injury during the hospital stay.^34^, ^35^, ^36^, ^37^ We have also demonstrated that impaired renal allograft function presents a risk factor for developing post‐COVID‐19 complications in general. All except one patient with acute allograft dysfunction and/or development of proteinuria already had chronic allograft dysfunction. Sepsis, hypoperfusion, exposure to nephrotoxic drugs, and injury to other organs may contribute to the development of acute tubular injury, while immunologic reactions associated with the SARS‐CoV‐2 infection may trigger glomerular alterations.

The immunomodulatory capacity of different viruses has been shown.^38^, ^39^ While some patients from our cohort developed de novo donor‐specific antibodies and even allograft rejection, others reactivated either CMV, EBV, or BK virus. These findings may indicate possible immunomodulatory action of SARS‐CoV‐2, emphasizing prompt further investigations while may be associated with significant clinical consequences, including the development of malignancies or acute rejections. This problem may exist in the general population but has not been investigated so far.

Coagulation abnormalities were frequent, most commonly shortened APTT and increased d‐dimers, while PT remained within the reference range. A normal PT with a shortened aPTT indicates the defect within the intrinsic pathway, with possible deficiency of factors VIII, IX, X, or XIII. In line with these findings, four patients developed thromboembolic complications. In contrast, one patient with a normal number of platelets developed spontaneous retroperitoneal bleeding. A growing body of evidence suggests that SARS‐CoV‐2 may induce different coagulation disorders associated with inferior outcomes. The most frequent pattern of coagulopathy in acute COVID‐19 patients includes increased levels of fibrinogen and D‐dimer, an increased PT and the aPTT, and a mild decrease in platelet count.^40^, ^41^, ^42^, ^43^ D‐dimer was most consistently associated with COVID‐19.^41^ The phenomenon of prolonged post‐COVID‐19 procoagulant state found in our cohort may be present in the general population as well and needs to be examined. These findings may have implications for understanding the effects of COVID‐19 on the development of thromboembolic complications. While the mechanism of this association remains unclear, SARS‐CoV‐2 infection has been associated with numerous physiologic alterations, including cytokine storm, which may influence the coagulation system,^44^ leading to venous thromboembolism.^45^ Normal platelet counts with procoagulant state indicated by shortened APTT and increased d‐dimers frequently found in our population may justify the use of acetylsalicylic acid in prophylactic regimens. It remains to elucidate if selected patients require more aggressive anticoagulation. However, several cases of spontaneous bleeding reported to date^46^ and one recorded in our group, highlighting the need for an individualized approach.

The complex interplay of the prothrombotic state caused by COVID‐19, anemia and the need to prevent cardiovascular complications required unprecedented treatment decisions. For this reason we were careful with introduction of erythropoiesis‐stimulating agents for treatment of anemia.

Decreasing the dose of tacrolimus during the acute COVID‐19 had a protective effect on developing laboratory complications in our cohort. It may be in contrast with previous findings of the potentially protective role for tacrolimus during the acute‐COVID‐19. A TACROVID trial showed that methylprednisolone and tacrolimus might have a beneficial effect in COVID‐19 patients with severe pulmonary failure and systemic hyperinflammatory syndrome, probably due to the ability of tacrolimus to inhibit both the SARS‐CoV‐2 replication and the secondary cytokine storm.^47^ A recently published meta‐analysis that included 202 solid organ transplant recipients concluded that receiving tacrolimus could benefit COVID‐19. However, their study included only 125 renal transplant recipients and was based generally on case reports or case series.^48^ While possibly beneficial during the acute COVID‐19 due to the anti‐inflammatory effect, tacrolimus may be associated with an additional endothelial injury^49^, ^50^ induced by SARS‐CoV‐2. Further studies are needed to clarify these results.

Six months after acute COVID‐19, most of our patients significantly improved and had no symptoms. However, many patients required rehospitalizations for severe complications. Some of them are hospital‐dependent, with complications arising one after the other. Reactivation of different viruses remains challenging. Longer follow‐up is needed to establish consequences, and those patients require close surveillance.

Long‐term follow up by pulmologists is necessary for patients with more pronounced respiratory symptoms, although most recovered rapidly.

This study has several limitations. First, the visit schedule was determined by the recovery from acute COVID‐19. It meant that we did not have the same time point for a check‐up for every patient, impacting the results. Second, baseline EBV DNA expression was not available, which may have led to an overestimation of EBV reactivations. Third, we had no baseline serum electrophoresis which may overestimate hypogammaglobulinemia as the post‐COVID‐19 complication. Fourth, we had no details for all patients regarding the intrahospital laboratory findings and treatment during the acute COVID‐19. Fifth, we had no results of SARS‐CoV‐2 serology. Finally, this is a single‐center study conducted in a tertiary referral center. Together with a relatively small number of patients in the study, this may potentially limit the generalizability of our results. However, this is the first study focused on the problem of post‐COVID‐19 in renal transplant recipients. It included hospitalized and ambulatory patients giving essential insights into clinical problems that occur even in patients without any symptoms at the initial presentation.

In conclusion, recovery from acute COVID‐19 is associated with different clinical and laboratory complications in the renal transplant population, regardless of the age or severity of initial symptoms. Complications were more frequent in patients with decreased glomerular filtration and patients with diabetes mellitus. A better understanding of the post‐COVID‐19 clinical course in this population may help direct actions to prevent major complications and mortality. All patients recovered from COVID‐19 should undergo long‐term monitoring for evaluation and treatment of complications. Further studies with long‐term follow‐up are needed.

## AUTHOR CONTRIBUTIONS

Nikolina Basic‐Jukic initiated the study, participated in study planning, designed data collection tools, and questionnaire, monitored data collection, prepared the statistical analysis plan, and drafted and revised the paper. Ivana Juric, Vesna Furic‐Cunko, and Lea Katalinic contributed to study planning, administered and monitored data collection, and revised the paper. Zrinka Bosnjak and Bojan Jelakovic participated in study planning, analysis, and revising the paper. Zeljko Kastelan contributed to study conception, study planning, data collection and monitoring, analysis and revised the paper. All authors reviewed and approved the final version.

## Data Availability

The data that support the findings of this study are available from the corresponding author upon reasonable request.
